# Dynamic arterial elastance predicts mean arterial pressure decrease associated with decreasing norepinephrine dosage in septic shock

**DOI:** 10.1186/s13054-014-0732-5

**Published:** 2015-01-19

**Authors:** Pierre-Grégoire Guinot, Eugénie Bernard, Mélanie Levrard, Hervé Dupont, Emmanuel Lorne

**Affiliations:** Department of Anaesthesiology and Critical Care, Amiens University Hospital, Place Victor Pauchet, Amiens, 80054 France; INSERM U1088, Jules Verne University of Picardy, Amiens, France

## Abstract

**Introduction:**

Gradual reduction of the dosage of norepinephrine (NE) in patients with septic shock is usually left to the physician’s discretion. No hemodynamic indicator predictive of the possibility of decreasing the NE dosage is currently available at the bedside. The respiratory pulse pressure variation/respiratory stroke volume variation (dynamic arterial elastance (Ea_dyn_)) ratio has been proposed as an indicator of vascular tone. The purpose of this study was to determine whether Ea_dyn_ can be used to predict the decrease in arterial pressure when decreasing the NE dosage in resuscitated sepsis patients.

**Methods:**

A prospective study was carried out in a university hospital intensive care unit. All consecutive patients with septic shock monitored by PICCO_2_ for whom the intensive care physician planned to decrease the NE dosage were enrolled. Measurements of hemodynamic and PICCO_2_ variables were obtained before/after decreasing the NE dosage. Responders were defined by a >15% decrease in mean arterial pressure (MAP).

**Results:**

In total, 35 patients were included. MAP decreased by >15% after decreasing the NE dosage in 37% of patients (*n* = 13). Clinical characteristics appeared to be similar between responders and nonresponders. Ea_dyn_ was lower in responders than in nonresponders (0.75 (0.69 to 0.85) versus 1 (0. 83 to 1.22), *P* <0.05). Baseline Ea_dyn_ was correlated with NE-induced MAP variations (r = 0.47, *P* = 0.005). An Ea_dyn_ less than 0.94 predicted a decrease in arterial pressure, with an area under the receiver-operating characteristic curve of 0.87 (95% confidence interval (95% CI): 0.72 to 0.96; *P* <0.0001), 100% sensitivity, and 68% specificity.

**Conclusions:**

In sepsis patients treated with NE, Ea_dyn_ may predict the decrease in arterial pressure in response to NE dose reduction. Ea_dyn_ may constitute an easy-to-use functional approach to arterial-tone assessment, which may be helpful to identify patients likely to benefit from NE dose reduction.

## Introduction

Vasopressors are an essential part of the early management of patients with circulatory shock [1]. Treatment of circulatory shock must include correction of the cause of shock and hemodynamic stabilization, primarily by fluid infusion and administration of vasoactive agents [2,3]. Finally, after achieving a minimal acceptable arterial pressure, providing adequate oxygen availability, and treating the cause of shock, the dose-reduction phase is necessary before discharge of the patient from the ICU. The purpose of this phase of treatment is to wean the patient from vasoactive agents and to promote spontaneous polyuria or induce fluid elimination to achieve a negative fluid balance [2]. The choice of appropriate treatment is based on a good understanding of the underlying pathophysiological mechanisms.

Norepinephrine (NE) is recommended in national and international guidelines as first-line hemodynamic support for septic shock [4]. In the SOAP study, norepinephrine was the vasopressor drug most commonly used (80% of cases), in combination with dobutamine in 30% of cases [5]. The use of NE is justified by its pharmacodynamic properties that induce arterial and venous vasoconstriction, allowing rapid correction of arterial pressure [6-8]. During the recovery phase of shock, as vasoplegia resolves after improvement of vasoreactivity, a theoretic risk of tissue hypoperfusion exists because of excessive vasoconstriction, especially when blood volume is not optimized. Decreasing the NE dosage is therefore an important step. However, few studies on the modalities of decreasing the NE dosage have been published [9]. The method used to decrease the NE dosage is often arbitrary and may unnecessarily prolong the potentially harmful use of this agent. No hemodynamic indicator predictive of the possibility of NE dose reduction is currently available at the bedside.

The ∆respPP/∆respSV ratio has recently been proposed as a dynamic indicator of arterial tone (arterial dynamic elastance: Ea_dyn_) [10,11]. Several authors have subsequently shown that this indicator can be used to assess vascular tone at the bedside, and that higher values were associated with the de-escalation of NE dose with fluid expansion [12,13]. Ea_dyn_ was also able to predict the hemodynamic response in mean arterial pressure (MAP) to fluid administration in hypotensive, preload-dependent patients [12]. Ea_dyn_ can therefore constitute a functional approach to the assessment of arterial tone similar to preload responsiveness parameters that are used to predict the hemodynamic response to a change in cardiac preload [12-14].

The primary objective of this study was to answer the following question: can dynamic arterial elastance be used to predict the decrease in arterial pressure induced by decreasing the NE dosage in sepsis patients? We also describe the effect of decreasing the NE dosage in resuscitated septic shock patients.

## Materials and methods

### Ethics

This study was approved by the Institutional Review Board (IRB) for human subjects (Comité de Protection des Personnes Nord-Ouest II CHU, Place V. Pauchet, 80054 AMIENS Cedex 1). Informed consent was waived, as the IRB considered the protocol to be an observational study. In our institute, the dosage of NE is decreased by 3.3 μg/min each hour for as long as MAP remains higher than 65 mm Hg. The indication to decrease the dosage of NE was left to the physician’s discretion. Only a one-step NE dose reduction was assessed in this study.

### Patients

A prospective, observational study was conducted at Amiens University Hospital intensive care unit over a period of 12 months (2012). Inclusion criteria were all consecutive patients with a diagnosis of severe sepsis or septic shock, according to the criteria of the Surviving Sepsis Campaign, treated with NE, for whom the attending physician decided to decrease the NE dosage, and who were monitored with a PICCO monitoring device [4]. Exclusion criteria were patients treated with epinephrine and/or dobutamine, arrhythmia, intraabdominal hypertension, and patients younger than 18 years. All patients had been sedated with continuous infusion of midazolam and sufentanil and were ventilated in volume-controlled mode.

### Hemodynamic parameters

An internal jugular vein central venous catheter was placed in all patients, and a thermistor-tipped arterial catheter (PV2024; Pulsion Medical Systems, Munich, Germany) in the femoral artery connected to a PICCO_2_ monitoring device was used to measure cardiac output (CO). Estimation of stroke volume by pulse-contour analysis was calibrated by transpulmonary thermodilution with injection of three 15-ml boli of cold saline. The mean value of three consecutive measurements was used for analysis of stroke volume (SV), CO, global end-diastolic volume (GEDV), and cardiac function index (CFI). If the difference between the three values was greater than 10%, two additional measurements were subsequently performed. Respiratory variations of pulse pressure (∆respPP) and stroke volume (∆respSV) were monitored by using PICCO_2_. Each value was the average of five consecutive measurements. Central venous pressure (CVP) and blood pressure were measured with a transducer zeroed at the level of the midaxillary line.

### Study protocol

The following clinical parameters were recorded: age, gender, weight, surgical/medical history, main diagnosis, and IGS2. Heart rate (HR), systolic arterial pressure (SAP), mean arterial pressure (MAP), diastolic arterial pressure (DAP), CVP, ∆respPP, ∆respSV, CO, systemic vascular resistance (SVR), and GEDV were recorded at baseline, with the patient adapted to the ventilator. A passive leg raising (PLR) test was performed to evaluate the effects on pulse-contour analysis of CO [15]. The dose of NE was decreased 10 minutes after PLR. After stabilization of hemodynamic variables, as assessed by the absence of variation of MAP by >10% over a 30-minute period, a second set of measurements with thermodilution (SAP, MAP, DAP, HR, CVP, CO, SVR, GEDV, ∆respPP, ∆respSV) was recorded. Dynamic arterial elastance (Ea_dyn_) was defined as the ratio of ∆respPP to ∆respSV. Static arterial compliance (C) (ml/mm Hg) was calculated as the ratio of SV to pulse pressure [15].

Ventilator settings were maintained at constant levels throughout the study period. Thermodilution calibration was performed before and after decreasing the dose of NE.

### Statistical analysis

A sample of 30 patients would be sufficient to demonstrate that Ea_dyn_ can predict a decrease in arterial pressure in response to decreasing the NE dosage with an AUC greater than 0.80, a power of 80%, an alpha risk of 0.05, and a beta risk of 0.2. Thirty-five patients were therefore recruited by taking into account the exclusion criteria. The distribution of the variables was assessed by using D’Agostino-Pearson test. Data are expressed as proportion (percentage), mean (standard deviation), or median (25^th^ to 75th percentiles), as appropriate. Nonresponders and responders were defined by MAP variation (expressed as a percentage) after decreasing the dose of NE. A positive response was defined as a ≥15% decrease in MAP [11]. The nonparametric Wilcoxon rank sum test, Student paired *t* test, Student *t* test, and Mann–Whitney test were used to assess statistical significance, as appropriate. Linear correlations were tested with the Spearman rank method. A receiver-operating characteristic curve (ROC) was established for SVR, arterial compliance, and Ea_dyn_. The test previously described by DeLong and colleagues was used to compare areas under the ROC curve (AUC) for each variable. Differences with a *P* value <0.05 were considered statistically significant. Medcalc 12.7.7.0 software (Mariakerke, Belgium) was used to perform statistical analysis.

## Results

Thirty-five patients with septic shock monitored by PICCO2 were included. The most common cause of septic shock was pneumonia and peritonitis (Table 1). Patients were included on day 6 (range, days 3 to 17) of admission to ICU. Thirteen (37%) of the 35 patients in whom the NE dosage was decreased were classified as arterial pressure responders because their MAP decreased by more than 15%.

**Table 1:** **Main patient characteristics on inclusion**

**Age (mean (SD), years)**	**65 (16)**
Gender (F/M)	13/22
SAPS 2 (mean (SD))	52 (12)
Etiology of shock, *n* (%)	
Pneumonia	15 (42)
Peritonitis	12 (34)
Endocarditis	3 (9)
Cholangitis	2 (6)
Salpingitis	1 (3)
*Clostridium* infection	1 (3)
Bloodstream infection	1 (3)
Respiratory parameters	
Tidal volume ((mean (SD), ml/kg of predicted body weight)	7.7 (1.1)
Respiratory rate (mean (SD), per minute)	21 (4)
Plateau pressure ((mean (SD), cmH_2_O)	23 (6)
Total PEEP ((mean (SD), cmH_2_O)	7 (4)
Left ventricular ejection fraction (%)	55 (9)

Values are expressed as mean (± standard deviation) or number (%). BMI, body mass index; SAPS 2, Simplified Acute Physiology Score 2.

In pressure-responder patients, the dosage of NE was decreased from 0.25 (0.14 to 0.58) to 0.23 (0.1 to 0.54) μg/kg/min^;^ in pressure-nonresponder patients, the dosage of NE was decreased from 0.38 (0.12 to 1.2) to 0.34 (0.09 to 1.2) μg/kg/ min. The median dose was not statistically different between the two groups (*P* = 0.43). The dosage of norepinephrine was decreased by 3.3 μg/min in all patients. At baseline, eight patients had ∆respPP and/or ∆respSV >15%. Apart from these patients, two were classified as pressure responders. Baseline CO variations in response to PLR were not significantly different between responders and nonresponders (1.6% (−4 to 8) versus 2.1% (−1 to 6), *P* = 0.87). SV decreased by more than 15% after decreasing the norepinephrine dosage in two patients.

**Figure 1:**
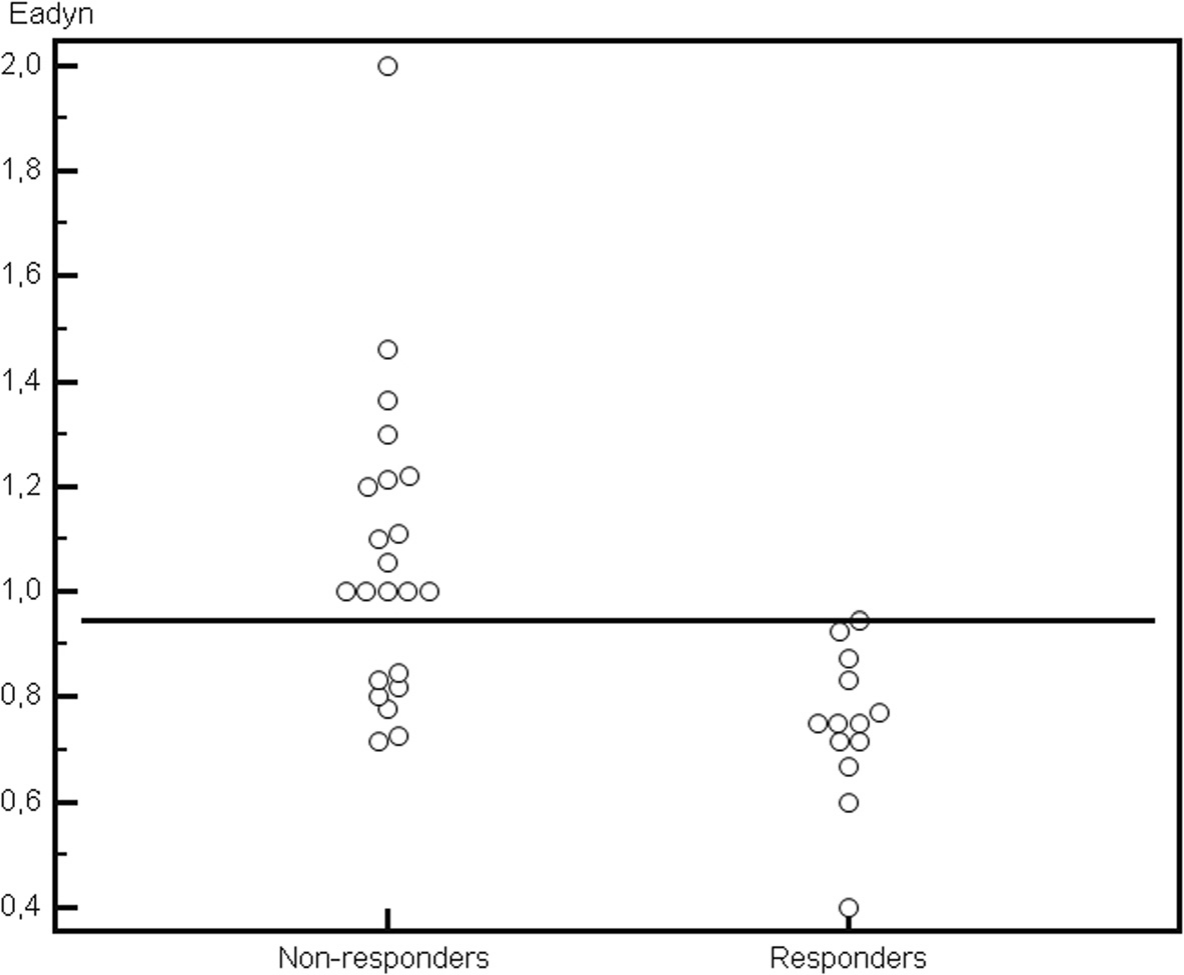
**Dynamic arterial elastance (Ea**_**dyn**_ **) in arterial-pressure responders and nonresponders.**

Baseline Ea_dyn_ was lower in patients in whom arterial pressure decreased after decreasing the dose of norepinephrine (Figure 1, Table 2). Arterial pressure and SVR decreased, and arterial compliance increased after decreasing the norepinephrine dosage in responders. SV, CO, EDGV, ∆respSV, ∆respPP, and CFI did not vary significantly in response to decreasing the norepinephrine dosage.

**Table 2:** **Cardiovascular variables in pressure responders and pressure nonresponders, expressed as median (25th to 75th percentiles) or mean (SD)**

	**Before**	**After**
**HR (beats/min)**		
Responders	84 (15)^a^	83 (15)^a^
Nonresponders	95 (17)	95 (18)
**SAP (mm Hg)**		
Responders	119 (12)	94 (11)^a, b^
Nonresponders	119 (13)	116 (14)^b^
**DAP (mm Hg)**		
Responders	56 (4)	45 (5)^a,^ ^b^
Nonresponders	59 (13)	57 (13)^b^
**PP (mm Hg)**		
Responders	63 (10)	50 (8)^a, b^
Nonresponders	60 (12)	58 (11)
**MAP (mm Hg)**		
Responders	77 (6)	61 (6)^a, b^
Nonresponders	79 (12)	77 (12)^b^
**CVP (mm Hg)**		
Responders	11 (5)	11 (5)
Nonresponders	11 (5)	11 (5)
Δ**respSV (%)**		
Responders	8 (6–15)	10 (8–15)
Nonresponders	10 (7–13)	10 (7–15)
Δ**respPP (%)**		
Responders	7 (4–11)	9 (5–12)
Nonresponders	10 (8–14)	10 (5–15)
**Ea**_**dyn**_		
Responders	0.75 (0.69-0.85)^a^	0.79 (0.67-1.04)
Nonresponders	1 (0.83-1.22)	0.9 (0.74-1.07)^b^
**Arterial compliance (ml/mm Hg)**		
Responders	1.2 (0.93-1.5)	1.5 (1.2-1.8)^a, b^
Nonresponders	0.98 (0.86-1.3)	1.1 (0.9-1.3)
**SV (ml)**		
Responders	70 (58–101)	67 (57–95)
Nonresponders	60 (53–75)	63 (53–77)
**CO (L/min)**		
Responders	6.2 (1.3)	6 (1.4)
Nonresponders	6.1 (1.8)	6.05 (1.8)
**GEDV (ml)**		
Responders	1,367 (361)	1,313 (334)
Nonresponders	1,371 (329)	1,375 (350)
**SVR (Dyn/s/cm** ^**−3**^ **)**		
Responders	891 (222)	703 (194)^a, b^
Nonresponders	963 (377)	942 (359)
**CFI (L/min)**		
Responders	4.7 (1.1)	4.7 (1.1)
Nonresponders	4.6 (1.4)	4.5 (1.3)

**CO**, cardiac output; **CFI**, cardiac function index; Δ**respSV**, respiratory Stroke Volume variation; Δ**respPP**, respiratory pulse pressure variation; **DAP**, diastolic arterial pressure; **Ea**: arterial elastance; **Ea**_**dyn**_: dynamic arterial elastance; **GEDV**, global end-diastolic volume; **HR**, heart rate; **MAP**, mean arterial pressure; **PLR**, passive leg raising; **PP**, pulse pressure; **SAP**, systolic arterial pressure; **SV**, stroke volume. ^a^ *P* < 0.05 between groups, ^b^ *P* < 0.05 within groups.

Ea_dyn_ was correlated with SAP, MAP, and DAP variations in response to decreasing the norepinephrine dosage (r = 0.41; *P* = 0.015; r = 0.47 *P* = 0.005; r = 0.49 *P* = 0.003), but no correlation was observed between SVR, SV variations, and a decreased dosage of NE (r = 0.004, *P* = 0.982; r = 0.26, *P* = 0.14, respectively). Ea_dyn_ was not correlated with norepinephrine dose (r = 0.23, *P* = 0.19).

**Figure 2:**
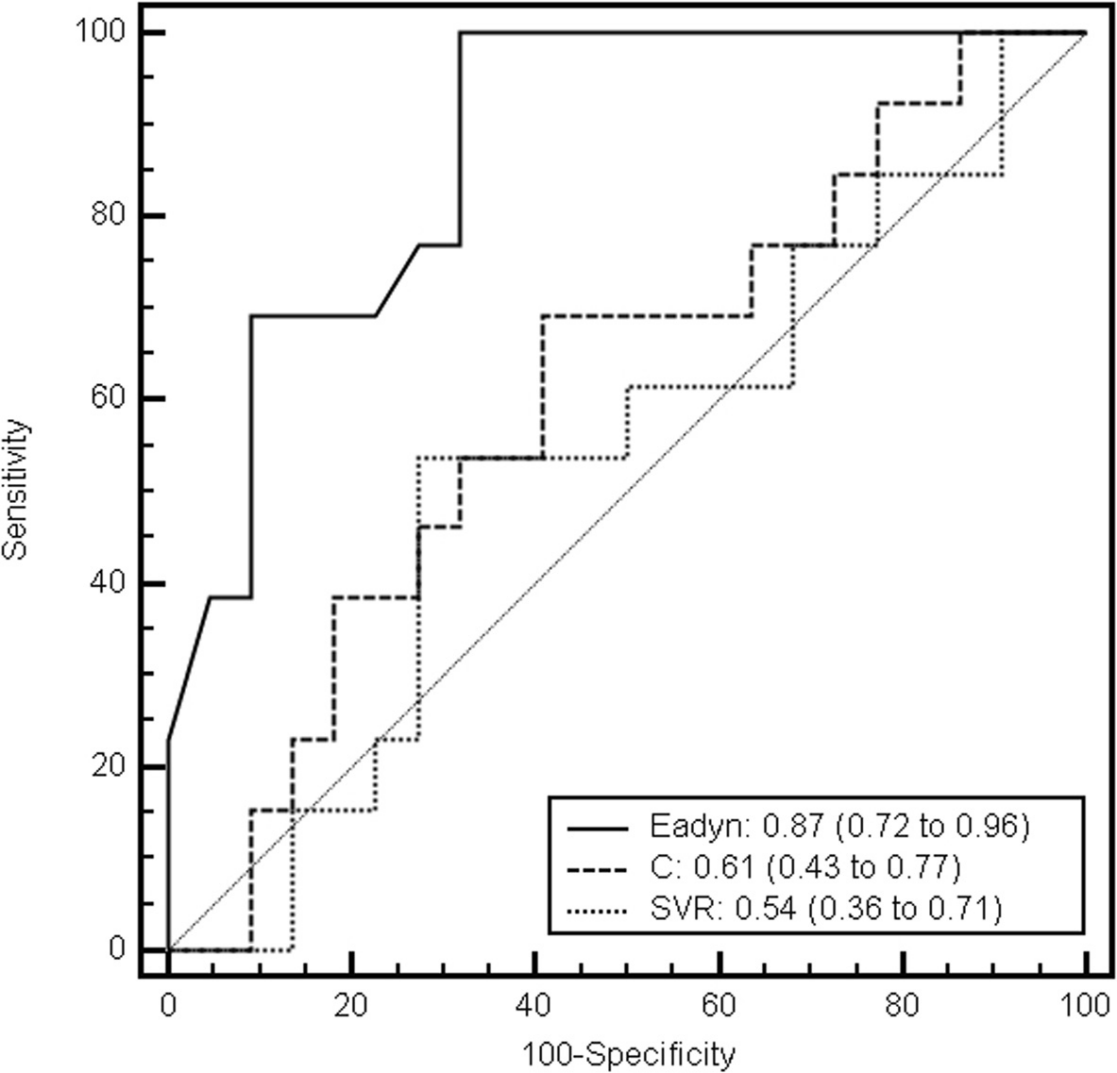
**Receiver operating characteristic curves to discriminate decrease in arterial pressure with decreasing doses of norepinephrine.** Ea_dyn_, dynamic arterial elastance; C, arterial compliance; SVR, systemic vascular resistance.

In the overall population, Ea_dyn_ predicted the decrease in arterial pressure with an AUC of 0.87 (95% CI, 0.72 to 0.96; *P* < 0.001) (Figures 1 and 2). The best cut-off was 0.94. Table 3 reports the various cut-off values for Ea_dyn_. Arterial compliance and SVR were not predictive, with an AUC of 0.61 (95% CI, 0.43 to 0.77; *P* = 0.32) and 0.54 (95% CI, 0.36 to 0.71; *P* = 0.41), respectively.

**Table 3:** **Accuracy of** Δ**respPP/**Δ**respSV ratio (dynamic arterial elastance) to predict decrease in arterial pressure**

**Cut-off value (%)**	**Sensitivity (%)**	**Specificity (%)**	**Positive likelihood ratio**	**Negative likelihood ratio**	**Positive predictive value (%)**	**Negative predictive value (%)**
0.94	100 (75–100)	68 (45–86)	3.14 (1.7-5.8)	0	65 (41–85)	100 (78–100)
0.83	77 (46–95)	73 (50–89)	2.82 (1.3-5.9)	0.32 (0.1-0.9)	63 (35–85)	84 (60–97)
0.77	69 (39–91)	86 (65–97)	5.08 (1.7-15.4)	0.36 (0.2-0.8)	75 (43–95)	83 (61–95)
0.7	23 (5–54)	100 (85–100)		0.77 (0.6-1)	100 (29–100)	69 (50–84)

## Discussion

This study, conducted on resuscitated sepsis patients, demonstrated that (a) Ea_dyn_ may be a functional indicator of arterial tone when decreasing the dose of NE that can differentiate patients in whom MAP is maintained from those in whom MAP decreases in response to a fixed dose reduction. An Ea_dyn_ less than 0.94 predicted a 15% decrease in MAP in response to decreasing the dose of NE. (b) The arterial pressure change associated with decreasing the dose of NE may be mostly due to the arterial vasoconstrictor (α-adrenergic) effect of NE.

No study has previously evaluated the predictive value of an indicator for NE dose reduction in septic shock patients. Ea_dyn_ has recently been proposed as a marker of arterial tone or, more specifically, a marker of arterial stiffness [14]. In postoperative cardiac surgery patients, vasodilator therapy decreased Ea_dyn_, whereas norepinephrine infusion increased this indicator [16]. In ICU patients, an Ea_dyn_ less than 0.9 was predictive of persistent arterial hypotension after fluid challenge, despite an increase in CO. [12]. In surgical patients, Ea_dyn_ was found to discriminate successfully responders and nonresponders to dose de-escalation with volume expansion [13].

In the present study, we demonstrated that Ea_dyn_ can identify patients in whom arterial pressure will decrease in response to NE dose reduction. Moreover, only Ea_dyn_ was predictive of the decrease in arterial pressure with a cut-off value close to previously published values [12,13].

Arterial pulse pressure results from the interaction between the blood volume ejected from the ventricle and the arterial system, which comprises several phenomena: stroke volume (SV), arterial wave reflection, wall stiffness, total peripheral resistance. The relation between arterial pressure and arterial volume is curvilinear [17]. In this context, Ea_dyn_ may indicate in which part of the curve the patient is situated. Ea_dyn_ may constitute a functional approach to arterial tone assessment in the same way as preload responsiveness indices that are used to predict fluid responsiveness to a change in cardiac preload [12,14]. Ea_dyn_ was lower in pressure responders, suggesting that SV variations induced low variations in PP due to the lower central vasomotor tone. Decreasing the NE dosage induced increased arterial compliance, which was even more marked in pressure responders_._ As discussed later, NE dose reduction induced only arterial α-adrenergic effects with no change in cardiac preload and CO. These effects may have been less marked than those observed in previous studies because of the limited decrease of the NE dose [8,18], but the effects were sufficient to alter vascular tone and the relation between PP and SV as assessed by arterial compliance, particularly in pressure-responder patients with a low Ea_dyn_. Because SV did not vary significantly, the decrease in arterial pressure (MAP and PP) was associated with changes in SVR and arterial compliance after the decrease of the norepinephrine dosage.

SVR was not significantly different between the two groups of patients and did not predict the subsequent course of arterial pressure. These results may be because SVR reflects a pressure difference between MAP and CVP, whereas, from a physiological point of view, the cardiovascular system comprises two pressure systems with a waterfall phenomenon [19]. SVR therefore does not reflect vascular tone, although it can be considered to be a component of vascular tone. At baseline, arterial compliance was probably not significantly different because of our small sample size, but appeared to be higher in pressure responders with low central vascular tone.

In the present study, the vascular effects of NE may depend on the sepsis patient’s underlying cardiovascular state. The vascular response observed differed from that reported in previous published studies [8,18,20]. Several explanations can be proposed for these differences. First, the patient’s cardiovascular status at the time of measurement: In contrast with other studies, we studied patients in whom NE dose reduction was initiated by the attending physician. In previous studies, preload indices were lower and CO variations with PLR were higher than in our study, suggesting that patients were insufficiently fluid-loaded [8,18,20]. Moreover, most of the patients included in our study also had ∆respPP values below the conventional cut-off for preload responsiveness: eight patients had ∆respPP over 15%. Among these patients, only two were classified as MAP responders and none significantly decreased their SV or increased their ∆respPP and ∆respSV values with NE decrease.

Another explanation could be that the dose of NE was not sufficiently decreased to induce any effects on venous return and cardiac preload [18,20]. The dosage of NE was decreased by a fixed dose that was lower than that used in studies evaluating effect of NE in sepsis patients. As multi-step NE dose reduction was not assessed in this study, we cannot exclude the possibility that these effects may be observed with more marked NE dose reduction. Decreasing the NE dosage resulted in a fall in arterial pressure with no significant change in cardiac preload and CO because patients were probably sufficiently fluid loaded (late phase of resuscitation). We observed an isolated decrease in arterial pressure with no decrease in CO, suggesting arterial α-adrenergic effects [8,16,17]. CFI, a surrogate marker of left ventricular function, did not decrease, suggesting no change in this index, whereas the decrease in arterial pressure was not related to α-receptors [21].

Our results must be interpreted cautiously, as up to 18% of patients had a false-positive response: despite a low Ea_dyn_, arterial pressure did not decrease by more than 15%. These results are in accordance with those reported by Hadian and colleagues [16], who observed that, in some patients, Ea_dyn_ was not correlated with changing doses of vasoactive drugs. Nevertheless, Ea_dyn_ had a high negative predictive value, showing that the upper Ea_dyn_ cut-off value of 0.94 is highly predictive of successful NE dose reduction.

Another explanation could be that the dosage of NE was not decreased sufficiently in these patients to induce any significant vascular effects. This indicator was not sufficiently specific to contraindicate NE dose reduction by physicians/nurses at the bedside.

Further prospective interventional studies using algorithms with Ea_dyn_ are needed to confirm the performance of this bedside indicator to adjust the NE dosage. Our sample size was small but the study was constructed to demonstrate the ability of Ea_dyn_ to predict changes of MAP associated with decreasing the NE dosage. Some of the differences observed for hemodynamic parameters might have reached statistical significance with a larger patient cohort, although the directional changes would unlikely be reversed. We studied a one-step NE dose reduction that was the same for the overall population. As no further decrease in the NE dosage was evaluated, we cannot draw any conclusions concerning the subsequent course of hemodynamic parameters and Ea_dyn_ in response to a more marked reduction of the NE dose. We studied MAP changes only due to changes in NE doses and not due to other therapeutic interventions (for example, dobutamine weaning). These results cannot be extrapolated to patients concomitantly treated with dobutamine, who may represent up to 30% of all sepsis patients.

Another limitation could be mechanical ventilation that alters the predictability of volume-responsiveness indices. The influence of tidal volume on Ea_dyn_ was probably marginal, as ventilatory parameters were kept constant during the study period. This relation remains constant and predictive, even during spontaneous ventilation, provided that ∆respPP and ∆respSV values are sufficiently large to define a slope [14,22]. Thermodilution was used as the reference method for CO measurement, but different CO results might have been obtained if another reference method had been used [23].

We used the PiCCO™ that does not report individual SV values on a beat-to-beat basis. ∆respSV and ∆respPP represent an average over 30 seconds. CO was calculated by using an algorithm based on the ventriculo-arterial coupling transfer function with thermodilution calibration for CO measurement. As a thermodilution calibration was performed before/after changing the NE dosage, we can assume that ∆respSV was calibrated to the change of vascular tone induced by NE.

## Conclusions

In this study, an Ea_dyn_ less than 0.94 was predictive of a decrease of arterial pressure in response to a decrease of the norepinephrine dosage in resuscitated sepsis patients. In contrast, no other hemodynamic variables were found to be predictive of a decrease in arterial pressure. Ea_dyn_ may constitute an easy-to-use functional approach to arterial tone assessment and may be helpful to identify patients likely to benefit from NE dose reduction. The high negative predictive value of this indicator can identify patients with a minimal risk of decreased arterial pressure. Further studies using algorithms with Ea_dyn_ are necessary to confirm the value and effectiveness of such an indicator at the bedside to adjust the NE dosage.

## Key messages

• Dynamic arterial elastance can be used to predict a decrease of arterial pressure associated with decreasing the norepinephrine a-dosage in sepsis patients.

• Systemic vascular resistance cannot predict the decrease of arterial pressure associated with decreasing the norepinephrine dosage in sepsis patients.

• The arterial pressure decrease associated with decreasing the norepinephrine dosage may be mostly due to the arterial vasoconstrictor effect of norepinephrine.

## Abbreviations

∆respPP: Respiratory pulse pressure variation
∆respSV: respiratory stroke volume variation
CFI: cardiac function index
CO: cardiac output
CVP: central venous pressure
DAP: diastolic arterial pressure
Ea: arterial elastance
Ea_dyn_: dynamic arterial elastance
GEDV: global end-diastolic volume
HR: heart rate
ICU: intensive care unit
MAP: mean arterial pressure
NE: norepinephrine
PLR: passive leg raising
PP: pulse pressure
SAP: systolic arterial pressure
SAPS 2: Simplified Acute Physiology Score 2
SV: stroke volume
SVR: systemic vascular resistance
